# Influence of Forest Therapy on Cardiovascular Relaxation in Young Adults

**DOI:** 10.1155/2014/834360

**Published:** 2014-02-10

**Authors:** Juyoung Lee, Yuko Tsunetsugu, Norimasa Takayama, Bum-Jin Park, Qing Li, Chorong Song, Misako Komatsu, Harumi Ikei, Liisa Tyrväinen, Takahide Kagawa, Yoshifumi Miyazaki

**Affiliations:** ^1^Korea Forest Service, Government Complex 1, 189 Cheongsa-ro, Daejeon 302-701, Republic of Korea; ^2^Forestry and Forest Products Research Institute, 1 Matsunosato, Tsukuba 305-8687, Japan; ^3^College of Agriculture and Life Sciences, Chungnam National University, 99 Daehak-ro, Yuseong-gu, Daejeon 305-764, Republic of Korea; ^4^Department of Hygiene and Public Health, Nippon Medical School, 1-1-5 Sendagi, Bunkyo-ku, Tokyo 113-8602, Japan; ^5^Center for Environment, Health and Field Sciences, Chiba University, 6-2-1 Kashiwanoha, Kashiwa, Chiba 277-0882, Japan; ^6^Finnish Forest Research Institute (METLA), P.O. Box 18 (Jokiniemenkuja 1), 01301 Vantaa, Finland

## Abstract

*Background*. Despite increasing attention toward forest therapy as an alternative medicine, very little evidence continues to be available on its therapeutic effects. Therefore, this study was focused on elucidating the health benefits of forest walking on cardiovascular reactivity. *Methods*. Within-group comparisons were used to examine the cardiovascular responses to walking in forest and urban environments. Forty-eight young adult males participated in the two-day field research. Changes in heart rate variability, heart rate, and blood pressure were measured to understand cardiovascular reactivity. Four different questionnaires were used to investigate the changes in psychological states after walking activities. *Results*. Forest walking significantly increased the values of ln(HF) and significantly decreased the values of ln(LF/HF) compared with the urban walking. Heart rate during forest walking was significantly lower than that in the control. Questionnaire results showed that negative mood states and anxiety levels decreased significantly by forest walking compared with urban walking. *Conclusion*. Walking in the forest environment may promote cardiovascular relaxation by facilitating the parasympathetic nervous system and by suppressing the sympathetic nervous system. In addition, forest therapy may be effective for reducing negative psychological symptoms.

## 1. Introduction

With an increasing interest in the health benefits of forest-oriented stimulations, considerable attention has been paid to forest therapy in many developed countries. The natural environment such as that of a forest is often considered to be an important factor in health promotion models [1] because it is closely associated with human health issues from the viewpoint of preventive medicine [2]. Artificial stimulations in urban environments have been considered to be a negative factor in modern health problems [3] in addition to physical inactivity [4] and chronic stress [5]. Many attempts have been made to improve human health through nature-related activities. In addition, exposure to the natural environment is known to reduce health inequality [6], increase perceived general health [7], and improve longevity of senior urban dwellers [8]. Physiological studies support that it has positive effects on the central nervous, autonomic nervous, and endocrine systems [9–14]. Immunology research shows that forest-related activity can increase human immune function by facilitating the activity of NK cells and anticancer proteins [15, 16]. Also, clinical trials prove that forest therapy programs can be effective for hypertension and non-insulin-dependent diabetic patients [17, 18]. In addition, psychological studies demonstrate that human beings have emotional affiliation to forest environments, which is effective for stress reduction, depression alleviation, and psychological relaxation [19–23].

Forest walking, walking with phytoncides in fresh forest air, beautiful scenery, and mild climate, is one of the most typical programs in forest therapy. A recent study has indicated that forest walking can improve self-rated health status and tends to decrease psychological stress in healthy individuals [24]. In addition, cognitive and affective benefits can be expected with forest walking in individuals with major depressive disorder [25]. Moreover, walking, the most common form of physical activity, is becoming increasingly important in the prevention of cardiovascular diseases [26–28]. However, till date, little evidence is available on the direct benefits of forest walking on cardiovascular reactivity.

Therefore, the aim of this study was to evaluate the short-term effects of forest walking on cardiovascular responses and to provide scientific evidence on the health outcomes of forest walking.

## 2. Methods

### 2.1. Participants

Forty-eight young Japanese adult males participated in the two-day field experiment. At the recruitment stage, only subjects free of previously diagnosed cardiovascular, allergic, or mental diseases were selected and those who were in poor physical condition were excluded from the study. Smokers and alcoholics were also screened at this stage. The mean age of the participants was 21.1 ± 1.2 (SD) years, and the mean body mass index was 21.3 ± 2.3 (SD) kg/m^2^ (Table 1). All participants were coded in the experiments. Vigorous physical activity, smoking, and alcohol consumption were prohibited throughout the experimental period. Before the beginning of the experiment, full explanation about the research purpose, the experimental procedure, and all measured indices was provided. Informed consent was obtained from all participants. Ethical approval for this study was obtained from the Ethics Committee of the Center for Environment, Health, and Field Sciences, Chiba University.

### 2.2. Physiological Markers

Heart rate variability (HRV) was measured to investigate physiological reactivity to forest walking and control activity by using a portable electrocardiograph (ECG) (Activtracer AC-301A, GMS, Tokyo, Japan) as an index of autonomic nervous activity [29]. Based on R-R interval frequency analysis, high frequency (HF) data were used as an index of parasympathetic nervous activity [30] and the low frequency/high frequency (LF/HF) ratio was used as an index of sympathetic nervous activity [31]. Heart rate was also investigated using R-R interval data. HRV and heart rate were recorded continuously while walking.

Systolic and diastolic blood pressure were also measured using a portable blood pressure monitor (HEM-1000, Omron, Tokyo, Japan). Blood pressure data were obtained three times and averaged before and after walking, respectively. All measurements were performed at field sites.

### 2.3. Questionnaires

Four different questionnaires were administered to investigate psychological reactions. These questionnaires have been used in many studies to investigate the psychological responses to natural environments [9, 10, 13, 14]. Subjective feelings of “comfortable-uncomfortable,” “natural-artificial,” and “soothed-aroused” were evaluated on the basis of a 13-scale questionnaire by using semantic differential (SD) techniques. The degree of the “refreshed” feeling was measured using a 30-question tool with a total score of 0–90 [32]. The shortened Japanese version of the Profile of Mood States (POMS) [33–35] questionnaire was used to measure mood states. Anxiety level was investigated using the Spielberger State-Trait Anxiety Inventory (STAI) questionnaire [36].

### 2.4. Stimuli and Experimental Design

Experimental stimuli included forest walking, self-paced walking in forest environments as part of the forest therapy program, and control stimuli included urban walking, self-paced walking activity in urban environments. Forest walking was performed in four different local areas of Japan in 2011, including Yoshino Town in Nara Prefecture (August 3-4), Akiota Town in Hiroshima Prefecture (August 9-10), Kamiichi Town in Toyama Prefecture (September 6-7), and Oita City in Oita Prefecture (September 13-14), to increase the generalizability of the results. The vegetation in the forest sites was dominated mainly by *Cryptomeria japonica*, *Chamaecyparis obtusa*, *Quercus serrata*, and *Acer palmatum*.

The forest walking course was selected in well-reserved forest zones, and the urban walking course was selected in urbanized zones in each local area. All walking courses were almost flat, and course length was set to be the same for forest and urban environments.

Each field experiment was performed for two days with generally fair weather, and 12 young adults participated in each experiment. All experimental procedures were the same throughout the four different areas. Twelve participants were randomly divided into two groups consisting of six individuals each; one group was assigned to the forest walking course and the other to the urban walking course. The groups switched the walking courses for the second day of the experiment to eliminate an order effect. Physiological measurements were performed individually. Because short-term effects of the walking activity were examined in this study, walking time was set at 12–15 min which was slightly varied depending on individuals. Energy expenditure for walking activity was also assessed using a triaxial accelerometer (Activtracer, GMS). Field experiments were performed at approximately the same time (10:00–12:00) in the forest and urban sites in all four study areas to eliminate the influence of diurnal changes in physiological rhythms.

### 2.5. Protocol

Physiological measurements and the questionnaires were pretested by all participants to aid comprehension and reduce errors in data collection at field sites. Prior to the experiments, three disposable electrodes were attached to the participants' chests to collect electrocardiogram data. In the morning, the participants travelled to the forest or urban experimental sites by car (45–70 min). The travelling time from the meeting room to the forest and urban sites was similar due to predefined travel courses. On arrival, the participants remained in the waiting room at each site and completed the first-session questionnaires. Each participant then moved to the walking course individually by foot or car, where they rested in the seated position for 10 min. Thereafter, they took a self-paced walk along the given course, during which heart rate data were recorded continuously. After walking, the participants rested for 5 min in the seated position. This was followed by blood pressure measurements and psychological tests using questionnaires. The experimental protocol was consistent throughout all experiments in all areas.

### 2.6. Data Analysis

Walking time was measured to investigate any differences in walking speed between the forest and urban courses. Heart rate was calculated by R-R data collected using ECG recorders during the walking activity. HRV data were analyzed by R-R intervals for a 1 min segment by using the maximum entropy method (MemCalc/win, GMS, Tokyo, Japan). The two major HRV spectral components, variances of the LF (0.04–0.15 Hz) band and the HF (0.15–0.4 Hz) band [37], were calculated. The HF values, which reflect parasympathetic nervous activity, and the LF/HF ratio, which reflects sympathetic nervous activity, were natural logarithm transformed for data analysis. Heart rate and HRV data during walking were compared between the forest and urban environments for 1 min. Blood pressure and questionnaire scores were also compared between the forest and urban environments. Two participants opted out of the experiment; thus, data from 46 participants were analyzed. All values are presented as mean and standard deviation. A paired *t*-test was used for the physiological data, and the Wilcoxon signed-rank test was used for the questionnaire data. Statistical analysis was performed using Excel 2010 (Microsoft Inc., Redmond, WA, USA), and subjective data were processed using StatView version 5.5 (SAS Institute Inc., Cary, NC, USA). The significance level was set at *P* < 0.05.

## 3. Results

### 3.1. Cardiovascular Reactivity

**Figure 1:**
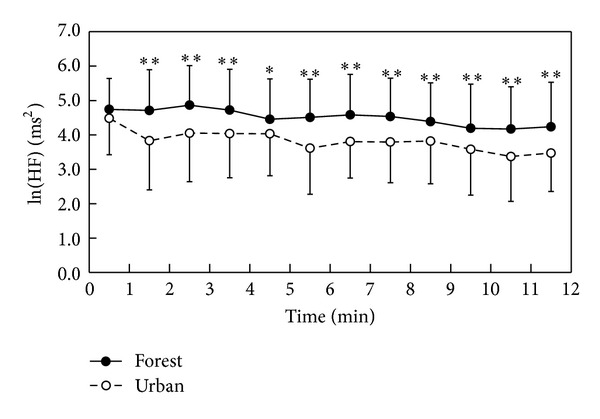
Temporal changes in ln(HF) values while walking in the forest and urban environments; *n* = 40–44, mean ± SD, paired *t*-test, *P < 0.05, **P < 0.01.

**Figure 2:**
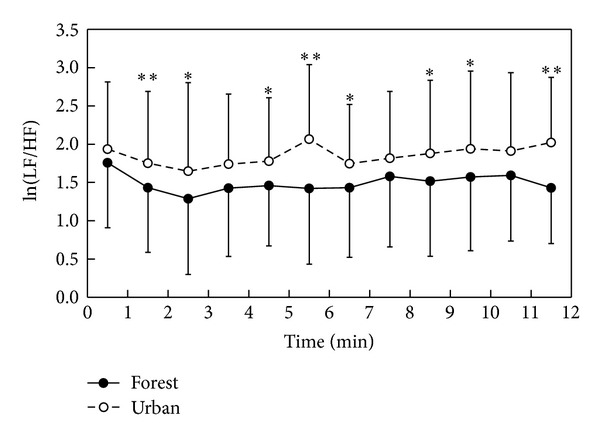
Temporal changes in ln(LF/HF) values while walking in the forest and urban environments; *n* = 40–44, mean ± SD, paired *t*-test, *P < 0.05, **P < 0.01.

We initially confirmed that there were no significant differences in the walking speed during forest walking and urban walking in all study areas. During stimuli, clear differences were found in the values of ln(HF) and ln(LF/HF) between forest walking and urban walking. The mean ln(HF) value during the total walking period was significantly higher in forest walking than in urban walking (4.4 ± 1.2 in forest and 3.8 ± 1.3 in urban; *P* < 0.01). For the 1 min segment analysis, significantly higher ln(HF) values were found in forest walking than in urban walking, except for the 0-1 min period (Figure 1). In contrast, the mean ln(LF/HF) value during forest walking was significantly lower in forest walking than in urban walking (1.5 ± 0.9 in forest and 1.9 ± 0.9 in urban; *P* < 0.01). The 1 min ln(LF/HF) value was significantly lower in forest walking than in urban walking, except for the periods of 0-1, 3-4, 7-8, and 10-11 min (Figure 2).

**Figure 3:**
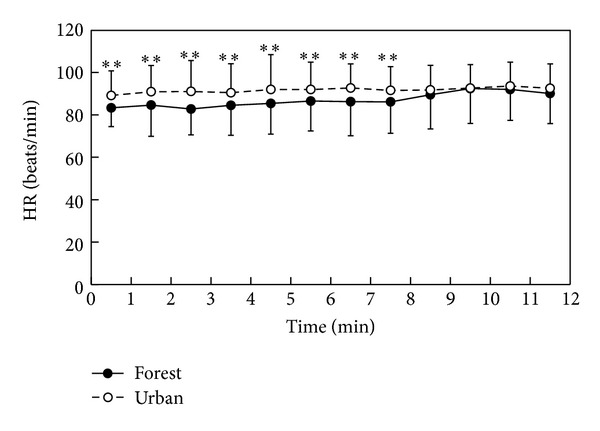
Temporal changes in heart rate while walking in the forest and urban environments; *n* = 42–45, mean ± SD, paired *t*-test, **P < 0.01.

Significant differences in heart rate values were observed between the forest walking and urban walking activities. In the 1 min analysis of heart rate during stimuli, significantly lower values were seen in forest walking than in the urban during 0–8 min period of stimuli (Figure 3). The mean heart rate value for the total stimuli period was significantly lower in forest walking than in the urban walking (87.2 ± 14.5 in forest and 91.8 ± 12.6 in urban; *P* < 0.01).

The mean systolic blood pressure after stimuli was lower in forest walking (114 ± 9.0 mmHg) than in the urban walking (116 ± 10.8 mmHg), but no significant difference was identified between the two. No significant difference was seen in diastolic blood pressure between the two after walking activities.

### 3.2. Questionnaire Analysis

**Figure 4:**
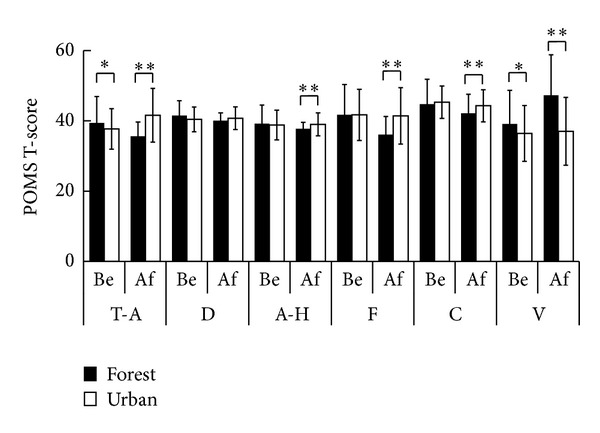
Comparison of the Profile of Mood States (POMS) subscale T scores between the forest and urban environments before (Be) and after (Af) walking. T-A: tension-anxiety; D: depression-dejection; A-H: anger-hostility; F: fatigue; C: confusion; V: vigor. *n* = 46, mean ± SD, Wilcoxon signed-rank test, *P < 0.05, **P < 0.01.

In the POMS questionnaire, significantly positive mood changes were observed after forest walking compared with those after urban walking (Figure 4). After forest walking, significantly decreased values were found in four negative subscales of tension-anxiety (T-A, 35.6 ± 4.0 at the forest site and 41.6 ± 7.6 at the urban site; *P* < 0.01), anger-hostility (A-H, 37.7 ± 1.8 and 39.0 ± 3.3; *P* < 0.01), fatigue (F, 36.1 ± 5.2 and 41.4 ± 8.0; *P* < 0.01), and confusion (C, 42.2 ± 5.4 and 44.3 ± 4.6; *P* < 0.01) in POMS. Although the T-A score before stimuli was significantly higher in forest walking than in urban walking, it significantly decreased after stimuli in forest walking than in urban walking. Vigor (V) scores, a positive subscale, were significantly higher in forest walking than in urban walking both before and after walking activities.

**Figure 5:**
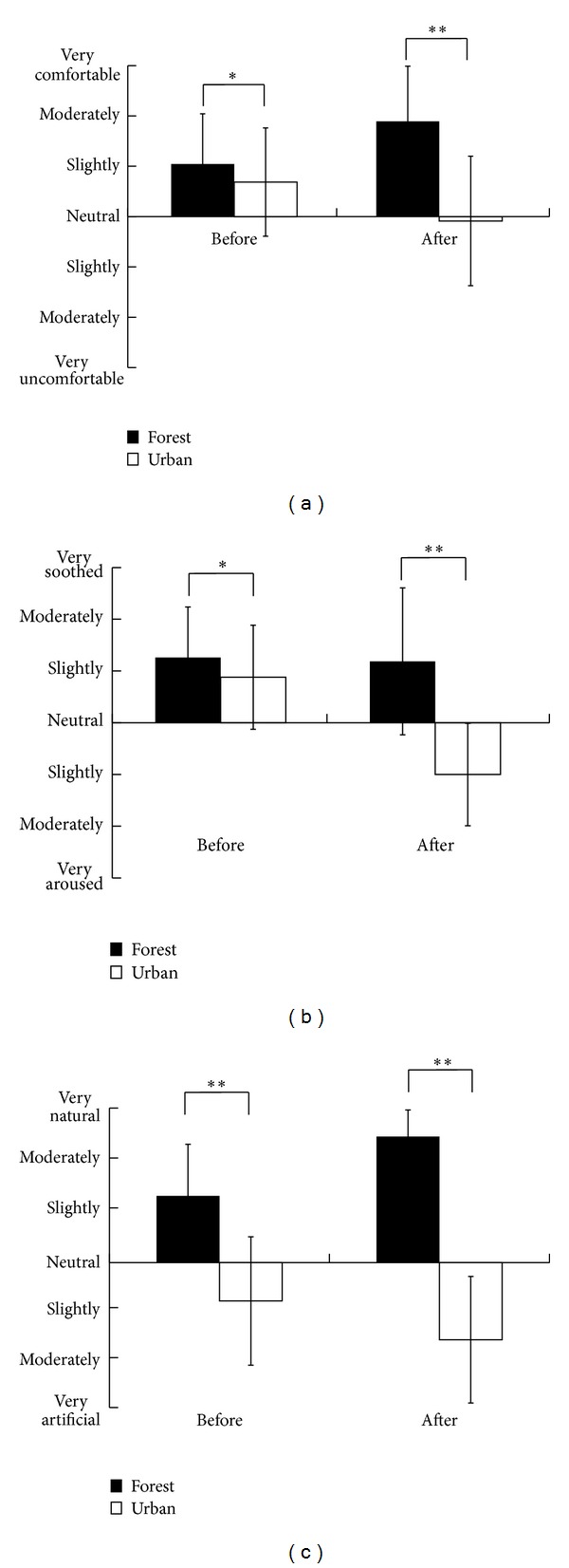
Subjective evaluation on “comfortable” (a), “soothed” (b), and “natural” (c) feelings before and after walking in the forest and urban environments; *n* = 46, mean ± SD, Wilcoxon signed-rank test, *P < 0.05, **P < 0.01.

Subjective evaluation using the SD method revealed that the participants felt more comfortable, soothed, and natural after forest walking than after urban walking both before and after activities (Figure 5). Significantly higher scores were observed for the “refreshed” feeling after forest walking (65.5 ± 10.7) compared with those for urban walking (50.4 ± 13.2; *P* < 0.01).

**Figure 6:**
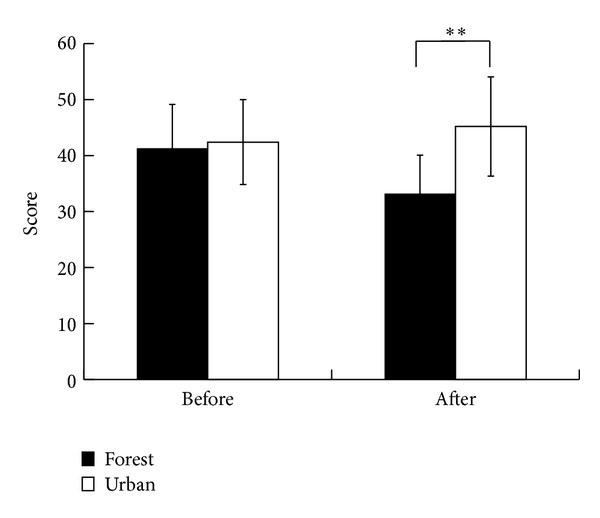
Comparison of the state anxiety level between the forest and urban environments before and after walking; *n* = 46, mean ± SD, Wilcoxon signed-rank test, **P < 0.01.

The Spielberger state anxiety levels were largely decreased after forest walking (33.2 ± 6.9) compared with those after urban walking (45.2 ± 8.9; *P* < 0.01), despite no significant difference between the two before walking (Figure 6).

## 4. Discussion

We performed field experiments in four different local areas to evaluate the physiological benefits of forest walking. Our data indicated that the forest walking program has a positive influence on cardiovascular relaxation. In the HRV analysis, forest walking was found to significantly increase parasympathetic nervous activity and significantly decrease sympathetic nervous activity compared with the urban walking. These trends in HRV response are often detected in meditation or yoga therapies [38–40]. The heart rate values were also significantly lower when walking in the forest compared with walking in urban environments, although no significant differences were observed in the mean calorie expenditure during the walking activity in the two environments. These results may reflect that physical activities in forest environments can facilitate cardiovascular relaxation, which is partly consistent with the results of previous field studies [9, 10].

Questionnaire analysis illustrated that forest environment can facilitate the psychological benefits of physical activity. Participants experienced less negative mood states such as tension-anxiety, anger-hostility, fatigue, and confusion and felt more comfortable, natural, soothed, and refreshed after forest walking compared with those after the urban walking. A previous study reported that physical activity reduces the anxiety level [41]; however, urban walking increased the mean level of state anxiety in this study (42.4 ± 7.6 before walking and 45.2 ± 8.9 after walking), which may indicate that artificial environments may reduce the positive health effects of physical activity [42].

The mechanism behind these health benefits of the natural environment remains to be a controversial issue. The natural environment is considered to be a catalyst for increasing physical activity which is a key factor for health promotion; however, a previous study reports that there is almost no relationship between the natural environment in living environment and physical activity [43]. Psychological studies demonstrated that a natural environment has positive effects on the reduction of mental stress and attentional fatigue [20–23]. In addition, natural environment can also be related to healthy behavior and social integration [44]. However, these relationships cannot give a sufficient explanation of the health benefit mechanism. The mechanism appears to be related more directly to human physiological responses to natural environment. The health benefit of forest-related activities may be explained by the recovery of homeostasis following acute and chronic stress. Forest-oriented stimulations facilitate the relaxation of central [13] and autonomic [9–12] nervous activities to suppress the secretion of stress hormones (cortisol, adrenaline, and noradrenaline) [9, 10, 16] and to recover decreased immune function [15, 16, 45]. These physiological responses to natural environment may interact with each other, resulting in positive health outcomes. This mechanism may partly explain the results of epidemiologic studies, which show the positive relationships between the natural environment and health parameters [6–8]. This concept is in accordance with Miyazaki's Nature Therapy Theory that human physiological functions have evolved to adapt to the natural environment; therefore, natural stimulation easily facilitates physiological relaxation [46].

Our findings indicated that physical activities in the forest environment can have positive effects on cardiovascular responses, which supports the fact that forest-related activity can be effective for facilitating homeostasis. However, further research with a bigger sample size is required to draw a generalized scientific conclusion.

## Figures and Tables

**Table 1:** Subject information.

Parameter	Value (Mean ± SD)
Total sample number	48
Sex	Male
Age (years)	21.1 ± 1.2
Weight (kg)	62.3 ± 7.6
Height (cm)	171.1 ± 4.9
BMI (kg/m^2^)	21.3 ± 2.3
